# The Methionine-Mitochondria Axis in Cancer: Metabolic Cross-Talk and Therapeutic Vulnerabilities

**DOI:** 10.1007/s40495-026-00481-y

**Published:** 2026-09-14

**Authors:** Md Salman Shakil, Matthew J. McBride

**Affiliations:** 1 Department of Chemical Biology, Ernest Mario School of Pharmacy, Rutgers University, Piscataway, NJ 08854 USA; 2 Rutgers Cancer Institute, New Brunswick, NJ 08901 USA

**Keywords:** Methionine, S-adenosylmethionine, Methylation, Cancer, Metabolism, Mitochondria

## Abstract

**Purpose of Review:**

This article describes the recent discoveries on how the amino acid methionine alters mitochondrial metabolism to support tumor function and growth. A detailed understanding of these mechanisms of cross-talk between the methionine cycle and mitochondria will empower the discovery and development of new metabolism-targeting cancer therapies.

**Recent Findings:**

Methionine and metabolites of the methionine cycle are increasingly appreciated to have both direct and indirect roles in regulating mitochondrial metabolism, which are critical for survival, growth, and treatment-resistance in tumors. Recent work has discovered multiple mitochondrial transporters that directly connect tumor use of methionine-derived S-adenosylmethionine (SAM) to mitochondrial function. Carnitine is synthesized from SAM-mediated methylation of lysine and is critical for tumor energy generation by fatty acid oxidation. Tumors depend on mitochondrial transport of SAM to support methylation reactions and oxidative phosphorylation. Purine synthesis is supported by mitochondrial one-carbon units from the folate cycle in tumors, which requires remethylation of homocysteine to form methionine to prevent folate trapping. Preclinical and clinical studies investigating both pharmacological and nutritional interventions are uncovering the mechanisms by which mitochondrial function depends on methionine metabolism. Further exploration in this area will define both the targets and specific interventions with the greatest promise for the treatment of cancer patients.

**Summary:**

Methionine metabolism influences many aspects of mitochondrial function, including energy generation, antioxidant defenses, and lipid composition. Understanding how tumors co-opt these processes and their dependence on the amino acid nutrient methionine provides an opportunity for new cancer therapies.

## Introduction to Methionine Cycle

**Fig. 1:**
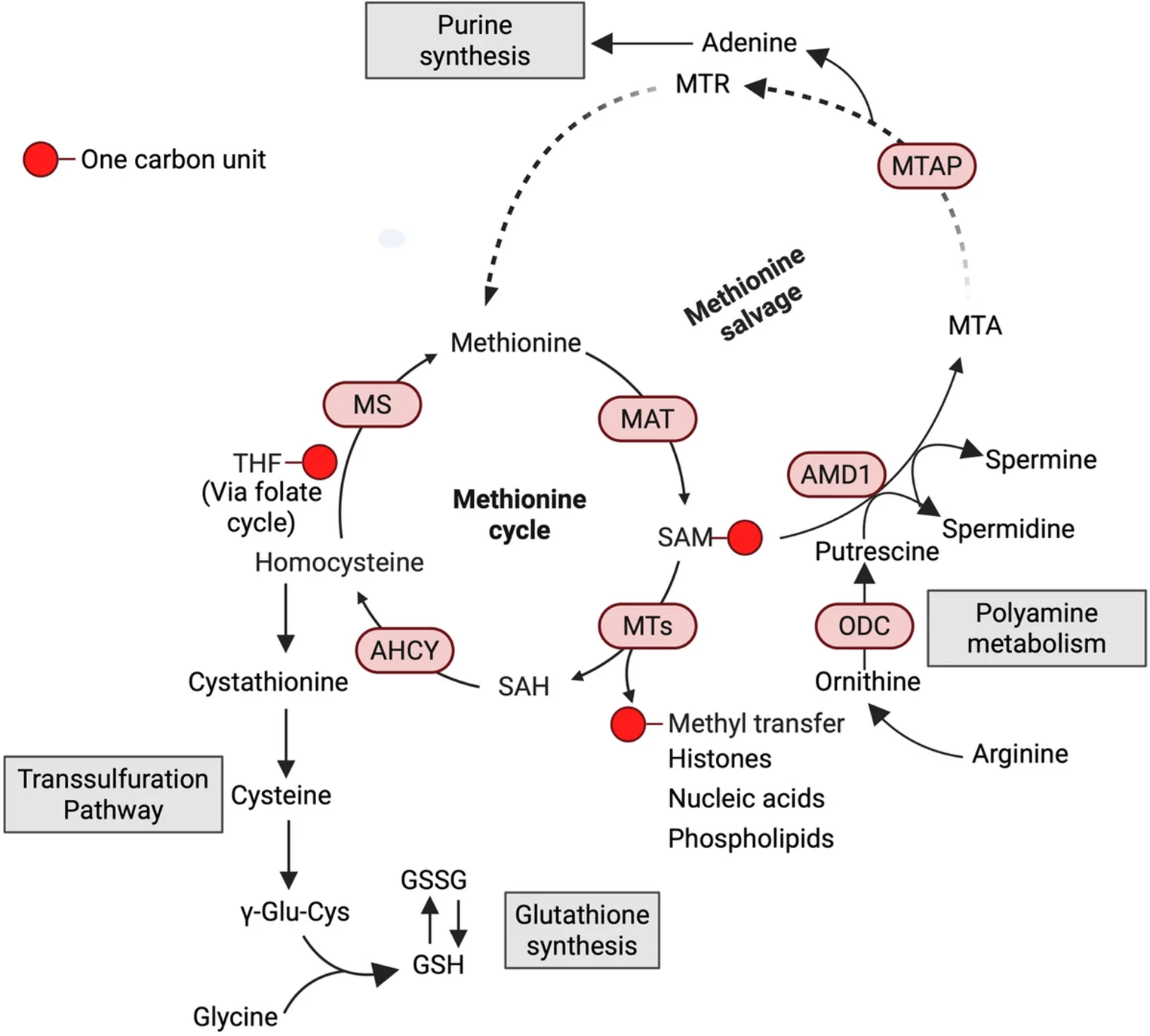
Methionine cycle and its interconnection to other metabolic networks. The methionine cycle supports multiple essential metabolic pathways, including methionine salvage, polyamine synthesis, transsulfuration for cysteine and glutathione production, and nucleotide biosynthesis. Methionine is converted into the universal methyl donor S-adenosylmethionine (SAM) by methionine adenosyltransferase (MAT). SAM serves as a substrate for methyltransferases, generating S-adenosylhomocysteine (SAH) during methylation reactions. SAH is then hydrolyzed by adenosylhomocysteinase (AHCY) to form homocysteine, which can either enter the transsulfuration pathway to support glutathione synthesis or be remethylated to regenerate methionine via methionine synthase (MS). Besides this, methionine can also be recycled from polyamine biosynthesis byproduct methylthioadenosine (MTA). The polyamine spermidine is produced by the SAM-dependent reaction of adenosylmethionine decarboxylase 1 (AMD1). Spermidine is converted to the final polyamine spermine by an additional SAM-dependent process. γ-Glu-Cys: γ-glutamyl-L-cysteine; GSH: reduced glutathione; GSSG: oxidized glutathione; ODC: ornithine decarboxylase; MTAP: methylthioadenosine phosphorylase; MTR, methylthioribose. Created with BioRender.com

Methionine is an essential amino acid catabolized and recycled via a series of biochemical reactions called the methionine cycle [1]. First, methionine is converted to S-adenosylmethionine (SAM) by the activity of three MAT proteins (MAT1A, MAT2A, and MAT2B) in mammals [2]. SAM is the universal methyl donor, which after methyl transfer to nucleic acids (DNA, RNA), proteins, or phospholipids, is converted to S-adenosylhomocysteine (SAH) [3–5]. SAH is then converted to homocysteine, which can either be remethylated to form methionine or converted to cysteine via the transsulfuration pathway (Fig. 1) [6]. Besides remethylation of homocysteine, methionine can also be regenerated by the methionine salvage pathway. The salvage pathway recycles methylthioadenosine (MTA), a byproduct of polyamine synthesis from decarboxylated SAM, back to methionine via the MTAP-mediated conversion to methylthioribose-1-phosphate [7, 8]. These biochemical reactions are intricately regulated and coupled with one-carbon metabolism [1, 8, 9]. Therefore, disruption at any single node can affect metabolite levels within different cellular compartments leading to cellular dysfunction, as commonly observed in cancer.

Cancer cells require exogenous methionine to support increased methionine cycle flux and SAM generated from the first step of methionine cycle is utilized in multiple methylation reactions including methylation of histones, nucleic acids, and phospholipids [10, 11]. Among the SAM synthesis enzymes (MATs, methionine adenosyltransferases), *MAT1A* is mainly expressed in the parenchymal cells (adult hepatocytes and bile duct epithelial cells), while *MAT2A* and *MAT2B* are expressed in non-parenchymal cells (e.g. hepatic stellate cells, and Kupffer cells) and extrahepatic tissues [12, 13]. In liver cancer cells, expression of *MAT2A* and *MAT2B* are increased, and progression of multiple cancer types depends on *MAT2A* overexpression [12, 14, 15]. High SAM levels alter expression of metabolic enzymes, increase global levels of the *N*^6^-methyladenosine (m^6^A) modification on mRNA, contribute to genome-wide hypermethylation of DNA, and increase phosphatidylcholine (PC) lipid synthesis to promote cancer progression, survival, and metastasis [14, 16–20]. Many studies have reported that targeting methionine cycle by dietary methionine restriction (MR), recombinant methioninase, or small molecule inhibitors of MAT2A enzyme suppresses cancer growth. Additionally, the combination of these methionine-targeting interventions with chemotherapeutic agents offers additive or synergistic therapeutic benefit (Table 1).

**Table 1:** Targeting tumor methionine metabolism offers therapeutic benefit

Intervention category	Treatment	Experimental model	Clinical phase	Treatment outcome	Reference
Single intervention	rMETase	Pancreatic cancer PDOX mouse model	Preclinical	Reduces tumor growth and overcomes acquired gemcitabine resistance	[21]
	rMETase and doxorubicin	USCS PDOX mouse mode	Preclinical	rMETase reduces tumor size and overcomes DOX resistance	[22]
	Dietary MR	B16F10 allograft mouse mode	Preclinical	Short-term MR reduces tumor burden and sensitizes to ferroptosis	[23]
	Dietary MR	BALB/C-nu/nu, NOD-SCID	Preclinical	Methionine depletion decreases osteosarcoma growth in PDO and PDX models	[24]
	Dietary MR	Metastatic solid tumors patients	Phase I	Therapeutic effect was not determined from this study; however, MR was well-tolerated in adult metastatic patients.	[25]
	AG-270 (MAT2A inhibitor)	MTAP-deleted solid tumor and lymphoma patients	Phase I	A 54%–70% decrease of plasma SAM levels in patients along with treatment associated side effects (elevation of liver function biomarkers, thrombocytopenia, anemia and fatigue) In progress (NCT03435250)	[7]
	ISM3412 (MAT2A inhibitor)	Advanced or metastatic solid tumors patients	Phase I	In progress (NCT06414460)	
	IDE-397 (MAT2A inhibitor)	MTAP-deleted advanced solid tumor patients	Phase I/II	Complete remission of bladder cancer in one patient and reduces non-small cell lung cancer burden by 33% in another patient In progress (NCT04794699)	
Dual intervention	rMETase and caffeine	SS PDOX mouse mode	Preclinical	rMETase reduces tumor growth and combination with caffeine enhanced the effect	[26]
	rMETase and tigatuzumab	Pancreatic cancer PDOX mouse model	Preclinical	Enhances tigatuzumab efficacy by increasing TRAIL-R2 expression	[27]
	SAM and IKE	OS-RC-2 xenograft mouse model	Preclinical	SAM treatment increases antitumoral activity of inhibited cystine import by IKE.	[6]
	Dietary CMR and RSL3	Syngeneic orthotopic murine glioma model	Preclinical	CMR sensitizes glioma to RSL3-induced ferroptosis and prolongs survival	[28]
	Dietary MR and radiation therapy	Endometrial cancer or lung cancer patients	Phase I	None (toxicities same as radiation therapy alone, however, MR was challenging, and unacceptable to most cancer patients)	[29]
	IDE-397 and AMG193 (PRMT5 inhibitor)	Patients with advanced MTAP-deficient tumors	Phase I/II	In progress (NCT05975073)	
	S095035 (MAT2A inhibitor) and TNG462 (PRMT5 inhibitor)	Advanced or metastatic MATAP null solid tumors	Phase I/II	In progress (NCT06188702)	

*rMETase* recombinant methioninase, *PDOX* patient-derived orthotopic xenograft, *PDO* patient derived organoid, *PDX* patient-derived xenograft, *USCS* undifferentiated spindle cell sarcoma, *SS* synovial sarcoma, *MR* methionine restriction, *CMR* cystine and methionine restriction, *SAM* S-adenosylmethionine, *IKE* imidazole ketone erasin.

## Roles of Methylation in Mitochondria

**Fig. 2:**
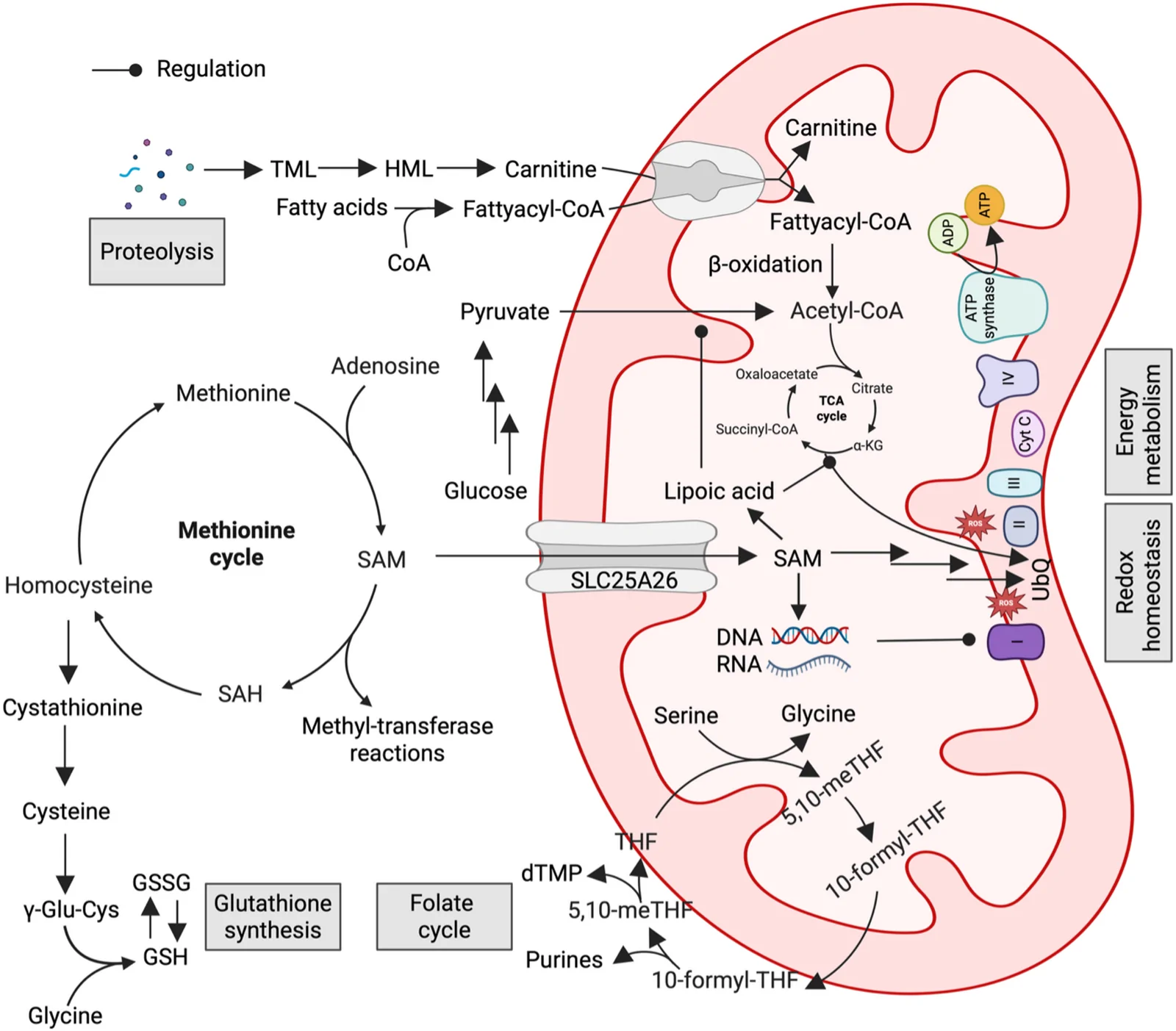
Integration of cytosolic methionine metabolism with mitochondrial transport, redox homeostasis, and energy production. Cytosolic SAM donates methyl groups during methyltransferase reactions, generating SAH and homocysteine. Homocysteine regenerates methionine or feeds into the transsulfuration pathway to synthesize GSH for redox control. SAM is also transported into the matrix via mitochondrial SAM carrier (*SLC25A26*) to support mtDNA/RNA methylation as well as lipoic acid and ubiquinone (UbQ) synthesis. Additionally, SAM is utilized for lysine methylation to form trimethyl-lysine (TML); subsequent proteolysis releases TML into the cytoplasm, where it acts as a precursor for carnitine synthesis. Carnitine assists fatty acid import into the mitochondria for β-oxidation to produce acetyl-CoA. Acetyl-CoA is also produced from the glycolytic intermediate pyruvate, a step regulated by lipoic acid, and subsequently enters the TCA cycle for oxidation reactions, which also require lipoic acid. Electron transfer from the TCA cycle to Complex I (regulated via mtDNA methylation dynamics) or Complex II is used for energy production. Excess electron accumulation at Complex III generates ROS. Simultaneously, mitochondrial folate metabolism shuttles one-carbon units to the cytosol for purine and dTMP synthesis. SAM: S-adenosylmethionine; SAH: S-adenosylhomocysteine; GSH: reduced glutathione; SLC25A26: solute carrier family 25 member 26; mtDNA: mitochondrial DNA; RNA: ribonucleic acid; UbQ: ubiquinone; TML: trimethyllysine; CoA: coenzyme A; TCA: tricarboxylic acid; Complex I: NADH-ubiquinone oxidoreductase (mitochondrial complex I); Complex II: succinate-Q oxidoreductase; Complex III: coenzyme Q-cytochrome c oxidoreductase; Complex IV: cytochrome c oxidase; ROS: reactive oxygen species; 5,10-meTHF: 5,10-methylenetetrahydrofolate; 10-formyl-THF: 10-formyltetrahydrofolate; dTMP: deoxythymidine monophosphate. Created with BioRender.com

SAM is synthesized in the cytoplasm and then approximately 30% is subsequently transported into mitochondria through the mitochondrial SAM carrier (mSAMC) (Fig. 2) [30]. SAMC is encoded by the *SLC25A26* gene which imports cytosolic SAM in exchange for mitochondrial SAH [31]. In many cancer types, mSAMC is aberrantly expressed leading to alteration of SAM transport and cellular compartmentalization [32]. SAM supports mitochondrial methylation by serving as the methyl donor for methyltransferases associated with rRNA, tRNA, and DNA methylation [4, 5]. SAM is required for mitoribosomal RNA processing and large subunit maturation. Consequently, mitochondrial translation was mildly reduced in 12-week-old *SLC25A26* conditional knockout (KO) mice [33]. The NOP2/Sun RNA methyltransferase 3 (NSUN3) modifies the cytosine base of mitochondrial tRNA^Met^ to 5-methylcytosine (m^5^C). This modified tRNA^Met^ helps to maintain oxidative phosphorylation and tricarboxylic acid (TCA) cycle metabolism in oral squamous cell carcinoma. Additionally, TCA cycle intermediates (e.g. succinate, fumarate, malate, α-ketoglutarate, citrate) and glycolytic metabolites (e.g. pyruvate) are depleted in NSUN3-deficient VDH15 primary patient-derived oral squamous cell carcinoma cells to suppress metastasis [5]. Recently, Menga et al. [4] reported that *SLC25A26* overexpression in CaSki cervical cancer cells increases mitochondrial SAM levels, promoting hypermethylation of mitochondrial DNA (mtDNA). mtDNA hypermethylation decreases the expression of Complex I subunit, leading to reduced mitochondrial ATP production and cytochrome c release. More importantly, mtDNA hypermethylation enhances sensitivity of CaSki cells to the chemotherapeutic agent cisplatin by increasing apoptosis [4].

Within mitochondria, SAM acts as a classical methyl donor for the synthesis of ubiquinone (UbQ, coenzyme Q10), and serves as a radical initiator during lipoic acid biosynthesis (Fig. 2) [31]. UbQ functions as a mobile electron carrier in the electron transport chain, shuttling electrons from complexes I and II to complex III. Additionally, it supports proton gradient formation through the Q-cycle to drive ATP synthesis [34]. Similar to UbQ, α-lipoic acid plays a vital role in energy metabolism. It is an essential cofactor for four mitochondrial enzyme complexes involved in energy metabolism, including pyruvate dehydrogenase (PDH), the glycine cleavage system (GCS), branched-chain 2-ketoacid dehydrogenase (BCKDH), and 2-ketoglutarate dehydrogenase (2-KGDH) [35]. α-lipoic acid stabilizes these multienzyme complexes to regulate their biochemical activity [36]. Interestingly, the PDH, BCKDH, KGDH enzymes can generate Reactive Oxygen Species (ROS) at higher basal rates than Complex I during the oxidation of TCA cycle metabolites [37]. Furthermore, SAM depletion through MR or *SLC25A26* KO decreases lipoylation of key mitochondrial proteins associated with TCA cycle and oxidative metabolism [38, 39]. MR reduces complete glucose oxidation and redirects acetyl-CoA and α-ketoglutarate toward anabolic pathways involved in amino acid synthesis, including arginine production [38]. Similarly, *SLC25A26* KO reduced lipoylation-dependent flux through PDH and 2-KGDH, limiting TCA cycle carbon influx and depleting aspartate as well as nucleotide pools [39].

## Energy Metabolism

Cytosolic SAM maintains the mitochondrial SAM (mitoSAM) pool and plays a critical role in cellular energy metabolism (Fig. 2). Depletion of mitoSAM reduces mitoSAM-dependent metabolites and disrupts complex I stability as well as iron-sulfur cluster (Fe/S) biosynthesis [40]. Fe/S clusters are essential cofactors for at least 48 enzymes involved in DNA synthesis and mitochondrial respiration, making Fe/S cluster metabolism a key link between oncogenic signaling and mitochondrial function [41, 42]. An upregulation of Fe/S cluster biosynthesis increases Complex I, Complex II, Complex III, aconitase, and cytochrome c, thereby promoting oxidative energy metabolism [43]. The small molecule Eprenetapopt suppresses Fe/S cluster biogenesis by inhibiting the cysteine desulfurase activity of NFS1. A decrease in Fe/S cluster levels impairs mitochondrial and cytosolic aconitase activity in OACM5.1 cells, indicating reduced TCA activity and energy production [41].

Carnitine primarily functions to shuttle long-chain fatty acids across the impermeable mitochondrial membrane for mitochondrial β-oxidation to meet the energy demands of cancer cells [44]. Trimethyllysine (TML) is the required metabolite precursor for L-carnitine biosynthesis. Lysine residues undergo post-translational methylation by SAM-dependent lysine methyltransferases to form TML, and free TML is released by proteolysis [45]. Alternatively, a small fraction of TML can also be obtained from the diet [45, 46]. L-carnitine is essential for transporting long-chain fatty acids into mitochondria for fatty acid oxidation (FAO). *SLC25A45* has recently been characterized as the mitochondrial transporter of TML that support carnitine synthesis [47]. *SLC25A45*-KO mice have reduced TML import into the mitochondria and thereby decreased carnitine levels, leading to impairment of FAO and dependence on carbohydrate metabolism [46]. Similarly, hepatic SAM during fasting functions as a metabolic sensor to control fatty acid oxidation. *MAT1A*-KO mice have reduced hepatic SAM levels, and during fasting these mice have increased lipid mobilization and elevated mitochondrial β-oxidation to support ATP production [48]. Furthermore, dietary MR (2 mg methionine/kg body weight per day) for 16 weeks significantly elevates fat oxidation and reduces hepatic lipid content in patients with metabolic syndrome [49]. Similarly, methionine depletion induced by L-*methioninase* in osteosarcoma cells reduces mitochondrial membrane potential and intracellular ATP levels while increasing ROS production. This methionine depletion caused decreased osteosarcoma growth in patient-derived organoid and patient-derived xenograft (PDX) models [24]. Similarly, siRNA-mediated knockdown of *MAT2A* or adenosylhomocysteinase (*AHCY*) in glioblastoma cells increases oxidative stress, disrupts cellular respiration, and reduces proliferation [50].

Methionine metabolism is also directly connected with glucose metabolism and cellular respiration [14, 51]. *MAT2A* overexpression in osteosarcoma cell lines increases aerobic glycolysis to support energy demand, cell growth and proliferation. Pharmacological inhibition of MAT2A enzymatic activity by the FIDAS-5 inhibitor significantly reduces aerobic glycolysis, lactate production and tumor growth, while increasing mitochondrial oxygen consumption and oxidative respiration [14]. Additionally, under glucose-deprivation conditions, AMP-activated protein kinase (AMPK) increases the interaction between MAT2A and programmed cell death protein 6 (PDCD6) in MS751 cervical cancer cells. This leads to increased PDCD6 stability due to MAT2A-mediated methylation, thereby suppressing apoptosis [52]. Overall, mitochondrial SAM supply and protein methylation directly influence both fatty acid oxidation through carnitine supply and cellular respiration (Fig. 2). Additional ongoing work is needed to elucidate which tumors are most dependent on SAM availability for nutrient utilization and energy generation, thereby enabling the targeting of this metabolic vulnerability.

## Interconnection of Methionine and Folate Cycles

The methionine cycle and folate cycle form a tightly interconnected one-carbon metabolic network compartmentalized between the cytosol and mitochondria [53]. One-carbon units that fuel the interconnected folate and methionine cycles originate from multiple metabolic sources, including serine, glycine, tryptophan, choline, and sarcosine metabolism [54]. Furthermore, metabolite exchange between these cycles acts as a key determinant of cellular methylation capacity, nucleotide synthesis, and redox balance [53, 55]. Mitochondria serve as a hub of one-carbon flux, where serine hydroxymethyltransferase 2 (SHMT2) converts serine to glycine and transfers one-carbon units to ultimately generate formate [41, 56, 57]. Formate is then exported and serves as a source of one-carbon units for cytosolic folate metabolism [58]. Mitochondrial one-carbon metabolism also provides bioenergetics (e.g. ATP), reducing power (e.g. NADH, NADPH) and glycine for reduced glutathione (GSH) synthesis [55]. This mitochondrial one-carbon output supports tumor progression: SHMT2-mediated mitochondrial serine catabolism sustains nucleotide synthesis and DNA damage repair in colorectal cancer cells resistant to the chemotherapeutic agent 5-fluorouracil (5-FU), and mitochondrial formate export drives fatty acid and cholesterol synthesis to promote glioblastoma cell invasion [59, 60]. Consistently, knockdown of methylenetetrahydrofolate dehydrogenase 1-like (MTHFD1L), a key enzyme involved in mitochondrial formate production, markedly suppresses lung metastasis in orthotopic 4T1 breast tumor-bearing mice [61]. However, serine’s mitochondrial fate has been shown to be tumor-context dependent. In fibrolamellar carcinoma, serine dehydratase (SDS)-mediated catabolism of serine to pyruvate, rather than one-carbon donation via SHMT2, supports TCA cycle flux and tumor viability, illustrating that serine can be diverted away from one-carbon/folate metabolism to fuel mitochondrial bioenergetics directly [62].

While mitochondria are the major site of one-carbon unit generation in tumor cells, cytosolic utilization of these one-carbon units supports critical cellular processes [58]. In the cytosol, folate-mediated one-carbon metabolism supports *de novo* purine synthesis, thymidylate production, and remethylation of homocysteine to methionine by methionine synthase (MS), linking folate availability directly to SAM production and downstream methylation reactions [1, 53, 63]. Because cells can import free methionine directly from the extracellular environment, MS activity is dispensable for maintaining intracellular methionine levels. Rather, MS is required to maintain SAM levels, nucleotide synthesis, and tumor cell proliferation [64]. This dependence is specific to the extracellular folate source: circulating folate in vivo is predominantly 5-methyl-tetrahydrofolate (5-methyl-THF), whereas standard cell culture media instead use folic acid, a non-physiological folate form. Under 5-methyl-THF (“physiological folate”) conditions, Ghergurovich et al. reported that MS knockout tumor cells trap folate disrupting purine biosynthesis, depleting nucleotide pools, and reducing tumor proliferation both in culture and in xenograft models [1]. Sullivan et al. independently confirmed this folate-source dependence and further showed that SAM levels, but not methionine levels, fall in MS-knockout cells under 5-methyl-THF. Because SAM synthesis by MAT2A consumes ATP, the authors proposed that this SAM decline reflects the ATP shortfall caused by impaired nucleotide synthesis, rather than methionine scarcity itself [64]. Neither study directly assessed mitochondrial function, leaving open how these cytosolic phenotypes relate to mitochondria. A recent study in *Mtr*^*+/−*^ C57BL/6J mice addressed this gap, showing that reduced MS activity causes accumulation of 5-methyl-THF, impairs cytosolic nucleotide synthesis, and increases uracil misincorporation into mtDNA. This leads to reduced liver mtDNA content by 25%, and causes a 20% decrease in the maximal oxygen consumption rate suggesting a direct link between cytosolic MS activity to mitochondrial genome integrity and respiratory capacity [65]. Recently, Cuyàs et al. showed that metformin modulates mitochondrial Complex I activity to alter one-carbon metabolism and increase the cellular SAM: SAH ratio, thereby promoting global DNA methylation (5-mC) in 4T1 mammary carcinoma cells [66]. This work showed that metformin redirects one-carbon units from SHMT2-dependent mitochondrial folate metabolism toward SAM synthesis coupled with an increase in Complex I-mediated energy production [66]. Together, these findings indicate the methionine and folate cycles are bidirectionally coupled to mitochondrial function: mitochondrial one-carbon output and Complex I activity shape cytosolic SAM availability, while cytosolic MS activity in turn safeguards mitochondrial genome integrity and oxidative capacity (Fig. 2). This bidirectional coupling represents a promising but still underexplored therapeutic target.

## Redox Homeostasis

Cancer cells naturally produce high levels of ROS as a result of the metabolic activity needed to support tumor growth. ROS are oxygen-containing chemical entities within cells encompassing hydrogen peroxide (H_2_O_2_), hydroxyl radical (OH·), and superoxide anion (O_2_^−^). Among the ROS-producing cellular compartments, mitochondria alone produce about 90% of cellular ROS [67]. In cancer cells, mitochondrial ROS are generated mainly through electron leakage from complexes I and III of the electron transport chain during oxidative phosphorylation [68, 69]. Through activity of proline dehydrogenase (PRODH), glycerol-3-phosphate dehydrogenase 2 (GPDH/GPD2), and dihydroorotate dehydrogenase (DHODH), electron transfer flavoprotein: ubiquinone oxidoreductase (ETF: QO/ETFDH) feeds electrons into the mitochondrial UbQ pool. When excess electrons accumulate, they can increase ROS production at Complex III and also drive reverse electron transport to generate ROS at Complex I [69]. Many standard-of-care therapies for solid tumors, including chemotherapy and ionizing radiation, further increase intracellular ROS levels [70]. ROS-derived oxygen radicals can covalently bind to and oxidize lipids, proteins, and DNA, leading to cell death [68]. However, tumor cells frequently adapt by upregulating antioxidant systems that neutralize ROS and maintain redox homeostasis, thereby developing resistance to therapy [70].

To counterbalance oxidative stress, cancer cells depend heavily on the antioxidant metabolite GSH [71, 72]. GSH is the major intracellular antioxidant and plays a critical role in detoxifying lipid peroxides and maintaining mitochondrial redox balance [72]. The synthesis of GSH requires three amino acids: glutamate, glycine, and cysteine [71]. Among these, cysteine is the rate-limiting substrate because its thiol group (R-SH) provides the reducing capacity necessary for ROS scavenging [73]. Cysteine can be imported directly or acquired through uptake of its oxidized dimer cystine via the cystine/glutamate antiporter system xc− (xCT). Once inside the cell, cystine is reduced to cysteine by intracellular reducing systems, including GSH and thioredoxin reductase 1 [71].

Methionine metabolism is closely linked to this antioxidant defense pathway. Cysteine is obtained from diet or produced from the methionine cycle intermediate homocysteine through the transsulfuration pathway (Fig. 1) [71]. Dietary MR or pharmacologic inhibition of SAM biosynthesis reduces homocysteine availability, thereby limiting transsulfuration and GSH production [74]. Decreased GSH levels disrupt redox balance and sensitize cancer cells to anticancer therapies. MR decreases GSH levels and sensitizes colorectal cancer cells to the chemotherapeutic agent 5-FU in PDX mouse model [9]. Additionally, cysteine biosynthesis from methionine is tissue specific, occurring mostly in liver and pancreas, yet was downregulated in tumors [75]. Under cystine deprivation conditions, methionine-derived SAM induces ferroptosis by increasing UbQ biosynthesis. Increased UbQ synthesis induces ferroptosis by increasing accumulation of lipid peroxides. More importantly, intraperitoneal SAM supplementation synergistically enhanced the antitumor activity of imidazole ketone erastin, which inhibits uptake of cystine, in OS-RC-2 renal carcinoma xenograft model [6]. MR reduces expression of acyl-CoA synthetase long-chain family member 4 (*ACSL4*), a potent ferroptosis promoting enzyme that catalyzes the conjugation of coenzyme A with long-chain polyunsaturated fatty acids (PUFAs), thereby limiting PUFA availability required for subsequent lipid peroxidation [76, 77]. This ACSL4-mediated incorporation of PUFAs into phosphatidylethanolamine (PE) or PC lipids promotes metastasis by increasing membrane fluidity and invasion [20]. Methionine also controls iron levels and sensitivity to ferroptosis by regulating lipid peroxidation clearance [23, 78]. Long-term consumption of L-methionine increases liver iron uptake and lipid peroxidation, with concomitant reduction of serum transferrin and iron levels [78]. As a metabolic precursor for cysteine and GSH synthesis, methionine metabolism through the methionine cycle and transsulfuration pathway can increase tumor tolerance to mitochondria-generated redox stress and represents a potential target for enhancing the efficacy of current cancer therapies.

## Lipid Metabolism

Mammalian mitochondria lack the machinery for phospholipid synthesis and therefore depend on the import of phospholipids from the endoplasmic reticulum. Among these phospholipids, PC is the most abundant lipid in mitochondrial membranes, forming about 50% of the outer membrane and 40% of the inner membrane [79]. The Kennedy pathway is the major route of PC lipid synthesis in mammalian cells where choline is used as the precursor for de novo PC lipid biosynthesis. In addition to supporting PC lipid supply, choline can also be oxidized in mitochondria to form betaine, which can donate a methyl group to homocysteine to form methionine in the liver and kidney [80]. In this process, betaine acts as an alternative methyl donor to 5-methyl-THF (a folate cycle intermediate), providing a metabolic shortcut to maintain methionine pools and preserve homocysteine homeostasis [81].

**Fig. 3:**
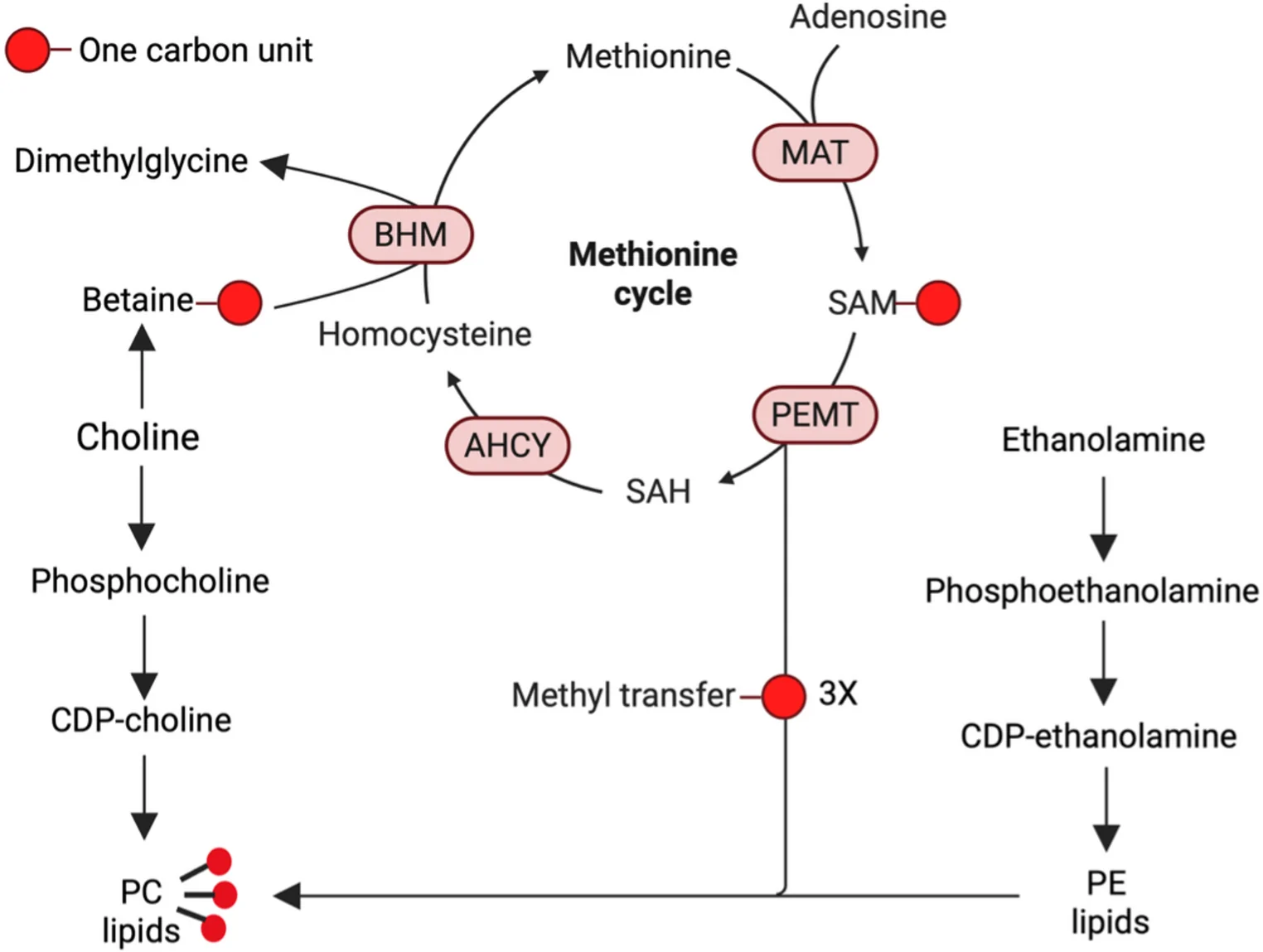
SAM is required for PC lipid synthesis by PEMT. PC and PE lipids are produced through the Kennedy pathway, and the two branches of the Kennedy pathway are connected through the PEMT pathway. Betaine, a choline derivative, can donate a one-carbon unit to homocysteine to regenerate methionine. PEMT: phosphatidylethanolamine-N-methyltransferase; PE: phosphatidylethanolamine; PC: phosphatidylcholine; SAM: S-adenosylmethionine; MAT: methionine adenosyltransferase; AHCY: adenosylhomocysteinase; BHM: betaine-homocysteine S-methyltransferase. Created with BioRender.com

Another route of PC lipid biosynthesis is the phosphatidylethanolamine-N-methyltransferase (PEMT) pathway where PE lipids undergo three rounds of SAM-mediated methylation (Fig. 3) [3, 82]. Consequently, methionine metabolism and SAM supply contribute to cellular control of phospholipid composition. In the liver, the PEMT pathway contributes up to 30% of PC biosynthesis and is enriched in PUFA-containing PC species [19]. Excess SAM generated from methionine-rich diet reroutes PE lipids for PC synthesis in the livers of mice. These PC lipids are then catabolized to produce diacylglycerol lipids leading to triacylglycerol accumulation and formation of lipid droplets [51]. Cells with active PEMT flux provide a major outlet for SAM utilization, which is necessary to maintain SAM homeostasis. Therefore, cells lacking PEMT activity rely on increased histone methylation, serving as a SAM sink, to prevent excessive SAM accumulation [3].

Besides phospholipids, cholesterol is another key component of cell membranes. Cholesterol is the major sterol that constitutes ~ 30–40% of mammalian cell membranes [83]. Cholesterol or cholesterol-derived metabolites promote cancer cell development, growth, proliferation, and immune suppression [84]. Additionally, metastatic tumor cells need high amounts of cholesterol to support tumor growth, and colonization of distant organ(s) [85]. SAM serves as a key regulator of cholesterol biosynthesis by maintaining cellular methylation potential. SAM depletion reduces DNA methylation of cholesterol-regulatory genes such as *SREBF1* and *LDLR*, leading to their upregulation and increased cholesterol biosynthesis in preadipocytes [86]. On the other hand, increased dietary methionine upregulates expression of key enzymes (e.g. 3-hydroxy-3-methylglutaryl coenzyme A reductase and cholesterol-7a-hydroxylase) for cholesterol biosynthesis in rat livers, leading to elevation of plasma and liver cholesterol levels [87]. Furthermore, there is a positive correlation between circulating plasma cholesterol and homocysteine levels [87, 88]. These findings indicate that methionine and SAM play tissue-specific roles in regulating cholesterol biosynthesis that require further investigation in tumors to understand how cholesterol targeting small molecules, such as statins, can be optimally leveraged for cancer therapy.

## Applications for the Clinic

Current efforts to develop clinical-grade MAT2A inhibitors are aimed at depleting SAM levels in cancers with 9p21.3 chromosomal deletions, which contain the coding region for the enzyme MTAP [89]. MTAP is a key enzyme in the methionine salvage pathway that converts MTA to methionine and adenine (Fig. 1) [90]. The frequency of homozygous MTAP deletion (*MTAP*^*−/−*^ genotype) occurs in approximately 10–15% of all solid tumors, ranging from 2% colorectal carcinoma to 55% in glioblastoma [91, 92]. Additionally, MTAP-deleted tumors frequently occur in male patients and older individuals and are associated with a shorter median time from diagnosis to comprehensive genomic profiling [92]. MTAP null cancer cells have increased accumulation of MTA which compete with SAM for binding to protein arginine N-methyltransferase 5 (PRMT5) [93]. Consequently, MTAP-null cancer cells are more vulnerable to SAM depletion, as the increased accumulation of MTA necessitates higher concentrations of SAM to maintain the catalytic activity of PRMT5 [94]. Suppression of PRMT5 activity decreases symmetrically dimethylated arginine (SDMA) residues on target proteins regulating mRNA splicing [7, 95]. A decrease in mRNA splicing prevents production of mature mRNA and impairs tumor proliferation by disrupting cell cycle progression [96]. Additionally, SAM depletion has been shown to disrupt the interaction of PRMT5 with its binding partner RIOK1 [97], thereby slowing tumor growth by altering ribosome biogenesis, cell cycle progression, gene expression, and metabolism [98].

Individual treatment with either the MAT2A inhibitor AG-270 (200 mg/kg) or the MTAP inhibitor MTDIA (20 mg/kg) was unable to reduce tumor burden in an HT-29 colorectal cancer (MTAP^+/+^) xenograft NOD/SCID mouse model [88]. However, combination treatment with the two agents reduced tumor growth by 66% compared with the vehicle control [91]. In another study, treatment of mice bearing pancreatic KP4 MTAP-null xenograft tumors with the MAT2A inhibitor AGI-25696 (300 mg/kg) via daily oral gavage for 33 days significantly reduced tumor burden without affecting body weight [89]. Similarly, oral administration of AG-270 (10–200 mg/kg) dose-dependently decreased SAM levels (33.4–81.1%) and suppressed tumor growth in the KP4 MTAP-null xenograft model. AG-270 was also well tolerated, with less than a 5% reduction in mean body weight [89]. Furthermore, a comparative study showed that SCR-7952 (3 mg/kg), a MAT2A inhibitor, exhibited greater antitumor activity than AG-270 (200 mg/kg) in reducing tumor volume in an HCT116 *MTAP*^*−/−*^ colorectal carcinoma xenograft mouse model. Moreover, the combination of SCR-7952 (1 mg/kg) and the PRMT5 inhibitor JNJ-64619178 (1 mg/kg) synergistically reduced tumor burden and decreases SDMA levels in the same mouse model [99]. These preclinical findings indicate that the antitumor activity of MAT2A inhibition is influenced by the MTAP status of the cancer. Consequently, multiple MAT2A inhibitors are currently in Phase I and II clinical trials for the treatment of MTAP-null cancers. Recently, Gounder et al. [7] reported the activity and tolerability of AG-270 in MTAP-deleted cancers in a Phase I clinical trial (NCT03435250). Once-daily or twice-daily AG-270 treatment for 28 days caused a 54%−70% decrease in plasma SAM levels in patients with MTAP-deleted solid tumors and lymphoma. Additionally, SAM depletion led to a reduction in SDMA residues. Common treatment-associated toxicities including increased liver function biomarkers, thrombocytopenia, anemia and fatigue were observed among patients [7]. In another Phase I trial (NCT06414460), the safety and tolerability of the MAT2A inhibitor ISM3412 are being investigated in participants with locally advanced or metastatic solid tumors. Beyond these Phase I trials, a Phase I/II clinical trial (NCT04794699) reported that treatment with the MAT2A inhibitor IDE-397 resulted in complete remission of bladder cancer in one patient, while the same treatment reduced non-small cell lung cancer tumor burden by 33% in another patient. Furthermore, the therapeutic benefit of the combination of IDE-397 and the PRMT5 inhibitor AMG193 is currently under investigation in a Phase I/II trial (NCT05975073) in patients with advanced MTAP-deficient tumors. Similarly, another on-going Phase I/II trial (NCT06188702) in patients with advanced or metastatic solid tumors harboring the *MTAP*^*-/-*^ genotype is exploring the therapeutic potential of the MAT2A inhibitor S095035 in combination with the PRMT5 inhibitor TNG462.

While current clinical efforts are targeting the unique vulnerability to reduced histone methylation caused by MTAP null tumors, there is an opportunity to leverage the development and favorable safety profiles of MAT2A inhibitors for patients with MTAP-positive tumors. By understanding and identifying the mechanisms by which tumors depend on SAM supply for mitochondrial functions, such as energy generation, these MAT2A-targeting therapies may be leveraged to improve cancer treatment for a broader patient population.

## Conclusions

Methionine metabolism regulates mitochondrial function and cancer cell metabolism through SAM-mediated epigenetic regulation, one-carbon metabolism, nucleotide biosynthesis, redox homeostasis, oxidative phosphorylation, FAO, and phospholipid remodeling. These metabolic adaptations directly and indirectly support tumor growth, survival, therapeutic resistance, and metastatic progression. Furthermore, the dependence of tumors on SAM-dependent methylation reactions and methionine turnover through the methionine cycle and methionine salvage pathway creates unique therapeutic vulnerabilities. Preclinical and clinical studies targeting methionine metabolism have demonstrated promising therapeutic potential, particularly in MTAP-deficient cancers. Finally, a deeper, cancer-specific understanding of the interactions among the methionine cycle, folate cycle, and mitochondrial metabolism will be essential for developing effective therapeutic strategies and rational combination therapies for cancer treatment.

## Key References

- Delaunay S, Pascual G, Feng B, Klann K, Behm M, Hotz-Wagenblatt A, et al. Mitochondrial RNA modifications shape metabolic plasticity in metastasis. Nature. 2022;607(7919):593-603. doi: https://doi.org/10.1038/s41586-022-04898-5.
  - This paper shows that methylation of mitochondrial mRNA supports oxidative phosphorylation to drive cancer metastasis.
- Rosenberger FA, Moore D, Atanassov I, Moedas MF, Clemente P, Végvári Á, et al. The one-carbon pool controls mitochondrial energy metabolism via complex I and iron-sulfur clusters. Science Advances. 2021;7(8):eabf0717. doi: https://doi.org/10.1126/sciadv.abf0717.
  - This paper establishes that mitochondrial SAM supply is needed for Complex I stability and biosynthesis of iron-sulfur clusters for cellular energy generation in both cancer and aging.
- Dias MM, King MS, Shokry E, Lilla S, Paul N, Thomason P, et al. SLC25A45 is required for mitochondrial uptake of methylated amino acids and de novo carnitine biosynthesis. Molecular Cell. 2025;85(21):4093-104.e8. doi: https://doi.org/10.1016/j.molcel.2025.08.018.
  - This work revealed that SLC25A45 is the mitochondrial transporter for trimethyllysine required for synthesis of carnitine and fatty acid oxidation.
- Gounder M, Johnson M, Heist RS, Shapiro GI, Postel-Vinay S, Wilson FH, et al. MAT2A inhibitor AG-270/S095033 in patients with advanced malignancies: a phase I trial. Nature Communications. 2025;16(1):423. doi: https://doi.org/10.1038/s41467-024-55316-5.
  - This Phase 1 trial of MAT2A inhibitor monotherapy establishes tolerability and proof of systemic SAM depletion in cancer patients.

## Data Availability

No datasets were generated or analysed during the current study.
